# A Uterine Polypus of Eighteen Years’ Growth Successfully Removed

**Published:** 1870-07

**Authors:** J. W. Brooks

**Affiliations:** Chicago


### Chicago Medical Journal.

A MONTHLY RECORD OF

*Medicine, Surgery, and the Collateral Sciences.*

EDITED BY J. ADAMS ALLEN, M.D., LL.D.; AND WALTER HAY, M.D.

VOL. XXVII.—JULY, 1870.—No. 7.

### Original Communications.

ARTICLE I.—*A Uterine Polypus of Eighteen Years Growth Successfully Removed.* By J. W. BROOKS, M.D., Chicago.

On March 28th, 1870, I was requested to visit and examine Mrs. ——, recently from St. P——, who gave me her history as follows: Age 52 years; married at 17 years; enjoyed uninterrupted good health from birth up to 1850, and prior to that time bore one child, now living. Early in 1850 she conceived again and without apparent cause miscarried, at the fourth month of gestation. Her general health continued good, but from this time the monthly molimen was markedly increased. In 1852 she again conceived, and miscarried at the fourth month.

From this period her health was impaired from repeated hæmorrhages, both at the month and during the interval. Four months from the last miscarriage a polypus, recently removed, came down during a hæmorrhage and presented at the vulva, occasionally interrupting the flow of urine. She was under the care of various medical gentlemen, scarce two agreeing as to the nature of the malady. About 1856 she visited Chicago for medical advice.

One gentleman gave no opinion; another, under whose care she was placed, pronounced it "the worst case of prolapsus uteri he had ever seen." She returned to her home after four months of treatment. At this time she plead for the removal of "the thing," as she called it, but no one approved of an attempt, and for twelve more years she continued to suffer from prostrating hæmorrhages, often confined to her bed for weeks, in consequence thereof. In 1866 the catamenia ceased. In December, 1869, after a copious and protracted hæmorrhage, she came again to Chicago, to seek further professional aid, and was examined by several members of the medical profession, who gave divers opinions. One, that it was an ossified tumor; another, that it was a fibrous tumor, involving the uterus, and, perhaps, appendices, etc., etc. She had not been able to urinate or defecate for years, except as she pressed up the polypus, and for twenty years had not been free from pain through the hips and in the lower part of the back.

After this relation the writer proceeded to investigate. The vulva were separated by an apparent fibrous body. Following that body with the index finger, a constriction was found about four inches from the external point, and at this place the great diagnostic difficulty was encountered, for above this the whole vagina was roofed over with the adventitious growth. Seizing it by the constricted portion, and drawing it downwards and backwards, I was able to carry the index finger under the arch of the pubes, and upward, until a rounded edge of a cup-shaped cavity (resembling an inverted toadstool) was reached. Carrying the end of the finger over this edge and drawing it downward, not less than one and a half gills of muco-purulent matter was emptied out, and this enabled me, as I thought, to enter a uterine sound within the cavity of the uterus, an inch or more, and felt sure the growth was attached to the inner, posterior and middle part of that organ. Subsequent examinations confirmed me in the opinion, and my friend Dr. Skeer, who was present at the last examination, coincided with me. I now gave to the patient the opinion that it was a uterine polypus, and thought it could be removed without very great danger to life. She instantly asked when I would do it, and the 14th of April was fixed for the operation. On the day named, assisted by Dr. Skeer, I protected a wire ecraseur with a gum catheter slipped over it, and carried the same, thus guarded, up to the constriction, on the posterior part of the polypus, and, by careful manipulation, was able to carry it over the flange and slip it into the cup, making careful and constant traction, and pressing with the finger, until it passed below the os uteri, around the pedicle in the bottom of the cup, then removing the catheter from the ecraseur, the ends of the wire were entered into the fenestra at the end of an ecraseur staff (a plate of which staff, with a spring chain, can be seen in the CHICAGO MEDICAL JOURNAL for June, 1867, and was first used in a case there narrated), the arch of the staff being carried under the pubes, upward and backward, until it reached the pedicle, when the wire was fastened to the slide, firmly, the loop of the ecraseur being nearly at a right angle with the staff. The polypus was soon removed, and with little pain attending the operation. The next difficulty was the removal of the polypus from the vagina, which required my full strength to bring the upper part through the vulva. The hæmorrhage for the moment was profuse, but a sponge saturated with a mixture of one part of Squibb's Perchlorid Ferri, and two parts of water, was placed directly over the bleeding vessels and soon checked it. A "T" bandage was snugly applied, and one-fourth of a grain of morphine was given with some brandy. She passed a comfortable night, and no unpleasant symptom followed. From the second day after the operation the vagina was washed out twice, daily, with a solution of Hyposulphite of Soda in Aqua Rosæ, for about two weeks.

The dimensions of this amorphous polypus, after the blood was removed, were as follows: Circumference, below the constriction, twelve inches; circumference at the constriction, nine inches; circumference above the constriction, eighteen inches; circumference, longitudinally, more than twenty inches. Weight, after the removal of the blood, one pound ten ounces. Estimated weight of blood removed, four ounces. The sinuses in the polypus were very large, and the structure was strictly fibrous. The shape was cylindroid from the lower part to and just above the constriction. From thence it was a reflex growth upwards and outwards in all directions, spreading like a flange over the crest of the pubes, linea ilio pectinea and promontory of the sacrum, forming a cup-shaped cavity, in the bottom of which a short pedicle, one and one-fourth inches in diameter, was found. In the cavity depended the uterus, surrounded by the adventitious growth. The expanded os and cervix uteri were drawn into the bottom of the cup. Herein consisted the difficulty in forming a correct diagnosis, and led a professional gentlemen of high standing to regard it as a fibroid disease of the walls of the uterus. On the ninth day after the operation, an examination with a speculum showed the parts healthy, and the os uteri contracting.

The lady at this writing, May 30th, 1870, is in perfect health. The polypus is preserved, at my office, 55 South Clark street, and any one desiring, can see it.

